# Efficient Trilayer Phosphorescent Organic Light-Emitting Devices Without Electrode Modification Layer and Its Working Mechanism

**DOI:** 10.1186/s11671-018-2668-1

**Published:** 2018-10-04

**Authors:** Xiaomei Peng, Haiwei Feng, Jiaxin Zhang, Shihao Liu, Letian Zhang, Wenfa Xie

**Affiliations:** State key Laboratory on Integrated Optoelectronics, College of Electronics Science and Engineering, Jilin University, Changchun, 130012 People’s Republic of China

**Keywords:** Organic light-emitting device, Trilayer, Impedance spectroscopy, Transient analysis

## Abstract

At present, numerous functional layers are introduced to improve the carrier injection and balance the carrier transport in organic light-emitting devices (OLEDs). Although it may be a good way to enhance the efficiency of devices, the introduction of functional layers would also result in extra process and long manufacture period. Actually, with the enrichment of material system, many appropriate materials could be chosen to share two or even more functions in OLEDs. Here, via impedance spectroscopy and transient electroluminescence analysis, di-[4-(*N*,*N*-ditolyl-amino)-phenyl] cyclohexane (TAPC) and 4,7-diphenyl-1,10-phenanthroline (Bphen) are demonstrated to serve as carrier injection and transport layers simultaneously. As a result, efficient trilayer OLEDs are achieved with comparable performances to conventional multilayer devices. Further studies have also been carried out to analyze the recombination and quenching mechanisms in devices. TAPC can block electrons effectively, while Bphen avoids the accumulation of holes. It makes carriers in emitting layer become more balanced, resulting in the reduction of efficiency roll-off.

## Background

It is known to all that organic light-emitting devices (OLEDs) have attracted considerable attention for solid-state lighting, full color displays, and so on. A good deal of functional layers, such as the anode modification layer (AML), cathode modification layer (CML), hole-blocking layer (HBL), and electron-blocking layer (EBL), have been introduced in the OLEDs to achieve high-efficiency and low turn-on voltage. The AML and CML are used to enhance the hole or electron injection, respectively [1, 2]. While the HBL and EBL can efficiently block the diffusion of the exciton from the luminescent layer into the transport layer [3]. Obviously, the multilayer structure becomes a frequently used way to improve device performance. However, since one more layer means an extra preparation process, excess function layers would also cause the long period and high cost that limit the development of their industrialization. With the improvement of the organic material system, some materials could play multiple roles in OLEDs due to their prominent properties. For example, deoxyribonucleic acid-cetyltrimetylammonium complex can act as hole-transporting layers (HTL) because of high hole mobility, meanwhile the low lowest unoccupied molecular orbital (LUMO) energy level makes it suit for the EBL [4]. 4,4′,4″-Tris (carbazol-9-yl)-triphenylamine (TCTA) is usually used to be HTL; besides, it can also serve as the host in emitting layer (EML) because of its high triplet energy [5, 6]. Hence, it is possible to simplify the structure without sacrificing the device performance by choosing appropriate material. However, few studies have been carried out on phosphorescent white OLEDs (PHWOLEDs) with simple structure [7, 8].

More recently, capacitance characteristics based on impedance spectroscopy (IS) measurement has been a widely used tool to investigate the physical mechanisms of OLEDs. The inflection point of the first peak in capacitance–voltage (C-V) curves has been reported to be corresponded to the turn-on voltage of OLEDs. It is also a very sensitive probe of carrier accumulation caused by the barrier in the interface of organic layers or the imbalance of charge injection and transport in devices [9–17]. Meanwhile, transient electroluminescence (EL) has also been the subject of intense technological as well as fundamental research, because transient EL studies have generated insight into the internal working mechanism in OLEDs. Transient EL is investigated by driving the devices with short, rectangular voltage pulses. The response times obtained from transient EL characteristics of devices provides an essential criterion for their application [18–28].

In this paper, via impedance spectroscopy and transient analysis, we confirm that di-[4-(*N*,*N*-ditolyl-amino)-phenyl] cyclohexane (TAPC) and 4,7-diphenyl-1,10-phenanthroline (Bphen) can be used to play multiple roles in OLEDs. Combined with bipolar transport material 4,4′-*N*,*N*′-dicarbazole-biphenyl (CBP), we fabricate efficient trilayer PHOLEDs. Obviously, the performance of trilayer OLED is comparable with the common multilayer OLEDs and even possesses better efficiency roll-off. It is interpreted by the mathematical model of exciton-quenching mechanisms. Subsequently, we focus on the carrier recombination and exciton-quenching mechanisms which occurred in monochromatic phosphorescent devices in order to proceed the further optimization of the structure. With the existence of Langevin and trap-assisted recombination in CBP-doped tris(2-phenylpyridine) iridium [Ir(ppy)_3_] and iridium (III) bis-(2-methyldibenzo-[f, h] quinoxaline) (acetylacetonate) [Ir(MDQ)_2_(acac)], two exciton-quenching mechanisms, i.e., triplet–triplet annihilation (TTA) and triplet–polaron annihilation (TPA), can be observed via the mathematical model.

## Methods/Experimental

### Device Fabrication

The small molecular organic materials used in our experiments are purchased from Luminescence Technology Corporation, i.e., TAPC, Bphen, 1,3,5-tri (m-pyrid-3-yl-phenyl) benzene (TmPyPB), and CBP. The phosphorescent dopant Ir(ppy)_3_, Ir(MDQ)_2_(acac) and bis [(4,6-difluorophenyl)-pyridinato-N,C^2^′] (picolinato) Ir(III) (FIrpic), and poly(3,4-ethylenedioxythiophene)-poly(styrene sulfonate) (PEDOT:PSS, PH8000) are obtained from Xi’an p-OLED. Thus, all materials and solvents are commercially available and used as received without further purification.

All devices are prepared on glass substrates covered with patterned indium tin oxide (ITO) stripes. Before film deposition, the ITO glass substrates are subjected to a routine cleaning process with rinsing in Decon 90, deionized water, drying in an oven, and finally treated in a plasma cleaner chamber for about 5 min. The PEDOT:PSS films are fabricated by spin coating from aqueous solution before depositing with the thickness to be approximately 40 nm, and then the PEDOT:PSS films are all annealed at 120 °C for 10 min.

All organic layers and cathode are evaporated by thermal vapor deposition using resistively heated tungsten filament and metal boats under high vacuum (~ 5 × 10^−4^ Pa) at a rate of 1–2 Å s^−1^ monitored in situ with a quartz oscillator. The cathode we used in our experiments is Mg:Ag (15:1) alloy, which is controlled independently by separate thin-film deposition monitors, so does the doping process in EML. Finally, four active areas of the devices on each substrate were 10 mm^2^, which is decided by the overlap between the anode and cathode via using a shadow mask [24, 25].

### Characterizations

Luminance–current density–voltage characteristics and spectra of unpackaged devices are measured simultaneously using Goniophotometric Measurement System based on spectrometer (GP-500, Otsuka Electronics Co. Osaka, Japan) in air at room temperature.

For the transient voltage decay measurement, high-speed switching diode (Philips, 1N4531) and arbitrary waveform generator (Rigol, DG5102) are connected with our devices in series orderly, and the transient voltage of the devices is recorded by a digital oscilloscope (Rigol, DS4054) after a consecutive signal averaging. In the transient EL measurement, the tested devices are driven by pulsed voltage with a pulse width of 1 ms using arbitrary waveform generator (Rigol, DG5102) as an electrical switch for driving tested devices and a trigger signal for starting the collection of EL signals. The transient EL response was detected and collected by using an avalanche photodiode (C30902) and time-correlated single-photon counting system.

The capacitance–voltage (C-V) characteristics are measured with an Impedance Analyzer (TH2829C, Changzhou Tonghui Electronic Co., Ltd., China) with oscillating amplitude of 100 mV and the repetition rate of 1 kHz. The range of dc bias applied by this setup allows sweeping from 0 to + 10 V [26].

## Results and Discussion

### Efficient OLEDs Simplified Without AML

**Fig. 1:**
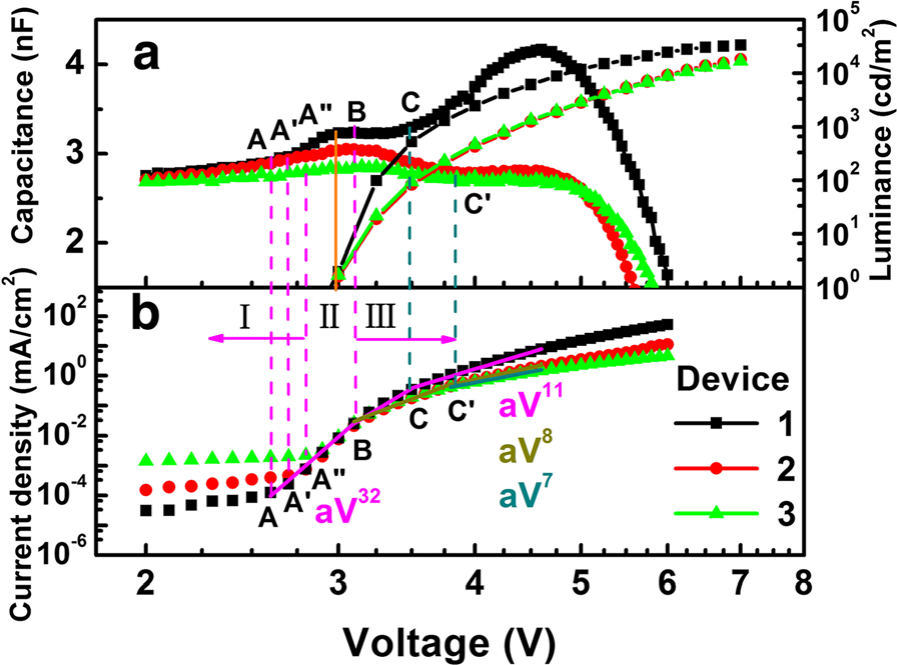
**a** Capacitance–voltage–luminance (C-V-L) characteristics of *D*_1_, *D*_2_, and *D*_3_. Orange solid line shows the turn-on voltage corresponding to the maximum of the first peak in their C-V characteristics. **b** Current density–voltage curves of the three devices in log–log scale, additionally divided into three regions marked by purple dashed lines marked I, II, and III. The current density (J) and voltage (V) conform to the relationship of $J\propto {aV}^m$

To get rid of AML, we choose TAPC as HTL in green phosphorescent OLEDs, because the highest occupied molecular orbital (HOMO) energy level is similar to the working function of ITO [5]. We perform contrastive experiments on an ITO/x/CBP:10 wt% Ir(ppy)_3_ (30 nm)/TmPyPB (50 nm)/LiF (0.5 nm)/Mg:Ag (120 nm) OLEDs, while the structure of *x* is TAPC (50 nm), MoO_3_ (3 nm)/TAPC (50 nm), and PEDOT:PSS (50 nm)/TAPC (50 nm), respectively. In order to differentiate the three devices, we mark them as *D*_1_, *D*_2_, and *D*_3_ in turns. Firstly, we investigate hole injection ability of these devices by analyzing their capacitance–voltage and current density–voltage–luminance characteristics. As we can see in Fig. 1a, the turn-on voltage of the three devices is about 3 V. It is relevant to the maximum of the first peak in their capacitance–voltage characteristics, indicating that it makes no difference to the turn-on voltage without AML in *D*_1_ [9–11]. Figure 1b shows the current density–voltage (J-V) characteristics of the three devices in log–log scale, we divide the J-V curves into three regions, (I) leakage or diffusion-limited current caused by Ohmic contact, (II) volume-controlled current with an exponential distribution of traps, and (III) volume-controlled current with partly filled traps [20]. The higher current density of device *D*_*3*_ at low applied voltage in region I may be attributed to the leakage current caused by the rough film morphology of solution-processed PEDOT:PSS films. In addition, the right shift of the turning point between region I and region II (from A to A”) presents the strongest carrier injection in *D*_1_, while the highest capacitance value of *D*_1_ indicates that more holes inject in the device and then accumulate in the interface or bulk [29]. Obviously, the interface of ITO/TAPC shows better hole injection ability. We can also find that the current density of *D*_1_ is larger than the values of the other two devices with the increase of the applied voltage. It may be attributed to the dipole layer generated between the ITO/TAPC interfaces. After introducing an extra AML, the intrinsic dipole layer is broken, resulting in the weaker injection ability among the two devices [10, 30]. In the reported references, the AML may be used to reduce the trap density which may have an impact on the stability of device [31]. For *D*_1_, the slope of the J-V curve in region III (*m* = 11) is larger than the values of *D*_2_ and *D*_3_ (*m* = 7, 8), the higher value of *m* always means higher trapping density [18]. The higher trapping density of device *D*_1_ may be attributed to the morphology change of TAPC film because of the lack of wetting layer, such as MoO_3_ or PEDOT:PSS. Moreover, the turning points C and C’ shown in Fig. 1 are relevant to the rapid increase of the injection of electron with the increase in bias voltage.

**Fig. 2:**
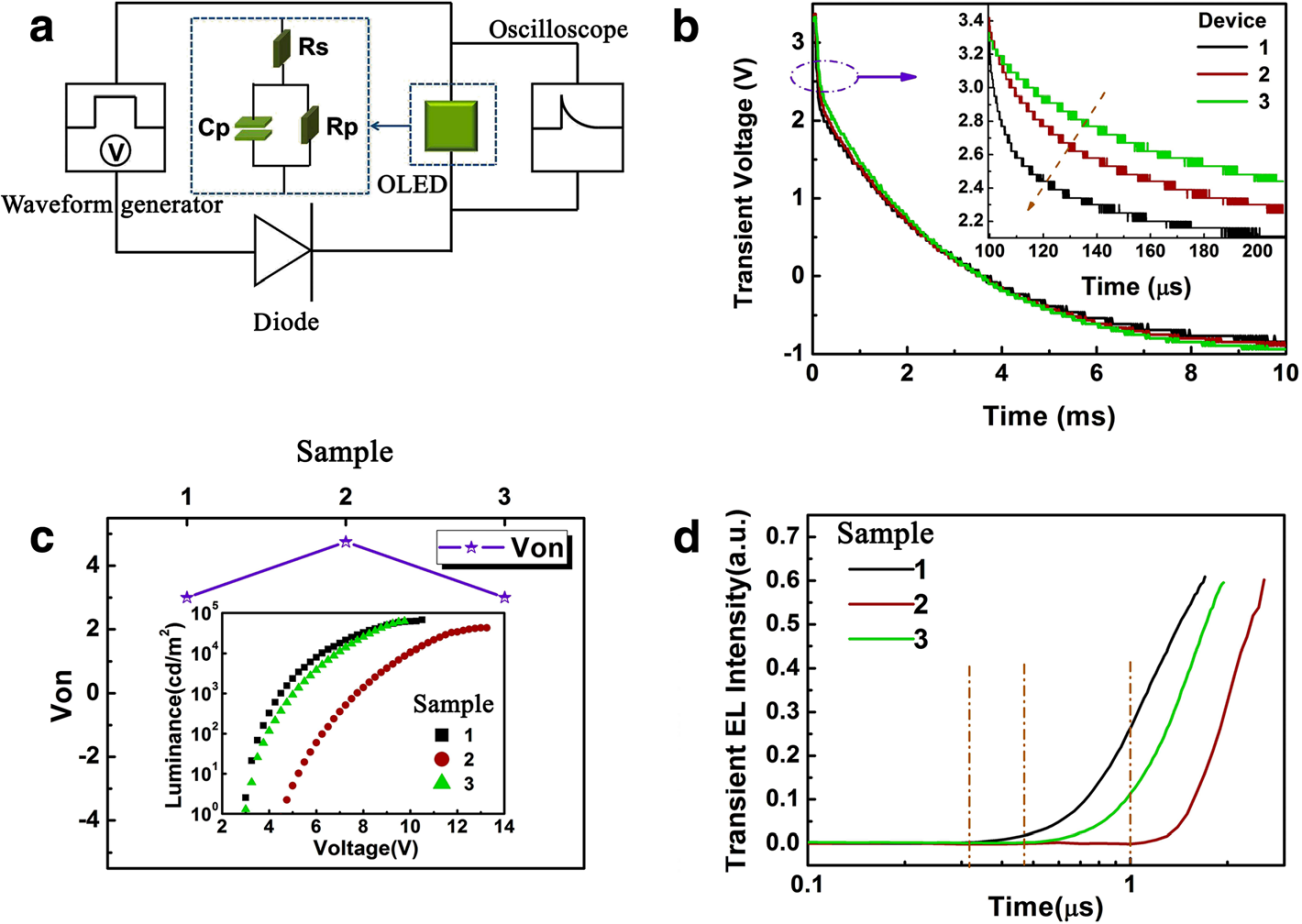
**a** Test circuit of transient voltage discharge characteristics measurement. **b** Time-resolved transient voltage decay characteristics at 5 V (*D*_1_, *D*_2_, and *D*_3_). [Inset: A fast decay time *τ*_1_ ≈ 100 μs. The orange dashed arrow shows different fall rates of devices]. **c** Turn-on voltage (*V*_on_) of *S*_1_, *S*_2_, and *S*_3_ [Inset: Luminance–voltage curves]. **d** Normalized time-resolved electroluminescence (EL) intensity of *S*_1_, *S*_2_, and *S*_3_ at 9 V. (The orange dashed line show the EL onset time of devices which is about 0.32 μs, 1.05 μs, and 0.48 μs, respectively)

A further study is carried out to research the carrier injection of the above devices by the transient voltage discharge characteristics. The test circuit is shown in Fig. 2a. Two response times are observed in Fig. 2b under the applied voltage of 5 V. The fast decay time *τ*_1_ is about 100 μs in the inset of Fig. 2b. Then, a followed slower decay *τ*_2_ is higher than one order of magnitude (*τ*_2_ is in the millisecond scale) [7]. The diode is regarded as a wire when the generator provides positive voltage. Charge carriers can transfer into the device easily, and then with the carrier injection barrier, there are a certain number of holes and electrons accumulated at the interface between organic layers, anode and cathode, respectively. The diode becomes infinite resistance inversely when the applied voltage turns to be negative. Charge carriers cannot reach the device, so the residual holes in the interface of ITO/organic layer can flow through organic layers and neutralize the remanent electrons diffused or drifted by space charges from cathode interface. Therefore, the downtrend of two response times, especially the *τ*_1_ are determined by the hole injection and transport ability of the organic layers in our contrast devices. It is obvious that the voltage of *D*_1_ falls at fastest rates, representing an excellent hole injection ability with the structure of ITO/TAPC merely. As the resistances of internal resistances in our samples reach to the magnitude of MΩ, the influence of the oscilloscope with 1 MΩ resistance cannot be ignored. That is why, only a little distinction can be seen in the three downtrends of *τ*_2_ [21, 22].

### Efficient OLEDs Simplified Without CML

Afterwards, we design a new contrastive experiment with the further simplification of ETL. As described in the reference reported by Scholz et al. [32], the metal–organic donor–acceptor adducts [Bphen+Ag]^+^ and [2Bphen+Ag]^+^ will form at the Ag-on-BPhen interface due to a self-doping effect. Our previous experimental results also indicated that these metal–organic adducts will improve the injection of electrons from Mg:Ag (15:1) to Bphen. Therefore, Bphen is chosen to be the appropriate experimental electron-transporting material here. The structure is ITO/TAPC (50 nm)/CBP:10 wt% Ir(ppy)_3_ (30 nm)/y/Mg:Ag (120 nm). The *y* is TmPyPB (50 nm)/LiF (0.5 nm), TmPyPB (50 nm), and Bphen (50 nm). *S*_1_, *S*_2_, and *S*_3_ are defined as the three samples, respectively. Figure 2c shows the turn-on characteristics of these three samples. It can be seen that *S*_3_ has the same turn-on voltage (*V*_on_ = 3 V) with *S*_1_, the luminance–voltage characteristics of *S*_3_ are also similar to those of *S*_1_ in the inset of Fig. 2c. So, we conclude that the simple structure in *S*_3_ owns great electron injection ability, which is on a par with *S*_1_. Moreover, we can investigate the carrier injection ability of the three devices by discussing the time-resolved behavior of the transient EL. The dashed lines in Fig. 2d show that the EL onset times of devices *S*_1_, *S*_2_, and *S*_3_ are about 0.32 μs, 1.05 μs, and 0.48 μs, respectively. The EL onset time is also called delay time (*t*_*d*_). It is composed of the injection time *t*_inj_ and transport time *t*_trans_. The larger threshold voltage *V*_th_ results directly in the longer *t*_inj_. Therefore, it is straightforward to prove that *S*_3_ can also possess excellent electron injection ability [23–25].

1 $$ {t}_d={t}_{\mathrm{inj}}+{t}_{\mathrm{trans}} $$

2 $$ {t}_{\mathrm{inj}}= RC\ln \left(\frac{V_{\mathrm{max}}}{V_{\mathrm{max}}\hbox{-} {V}_{\mathrm{th}}}\right) $$

3 $$ {t}_{\mathrm{trans}}=\frac{d_e}{\left({\mu}_e+{\mu}_f\right)E} $$

### Performance Comparison Between Simple Trilayer and Multilayer OLEDs

**Fig. 3:**
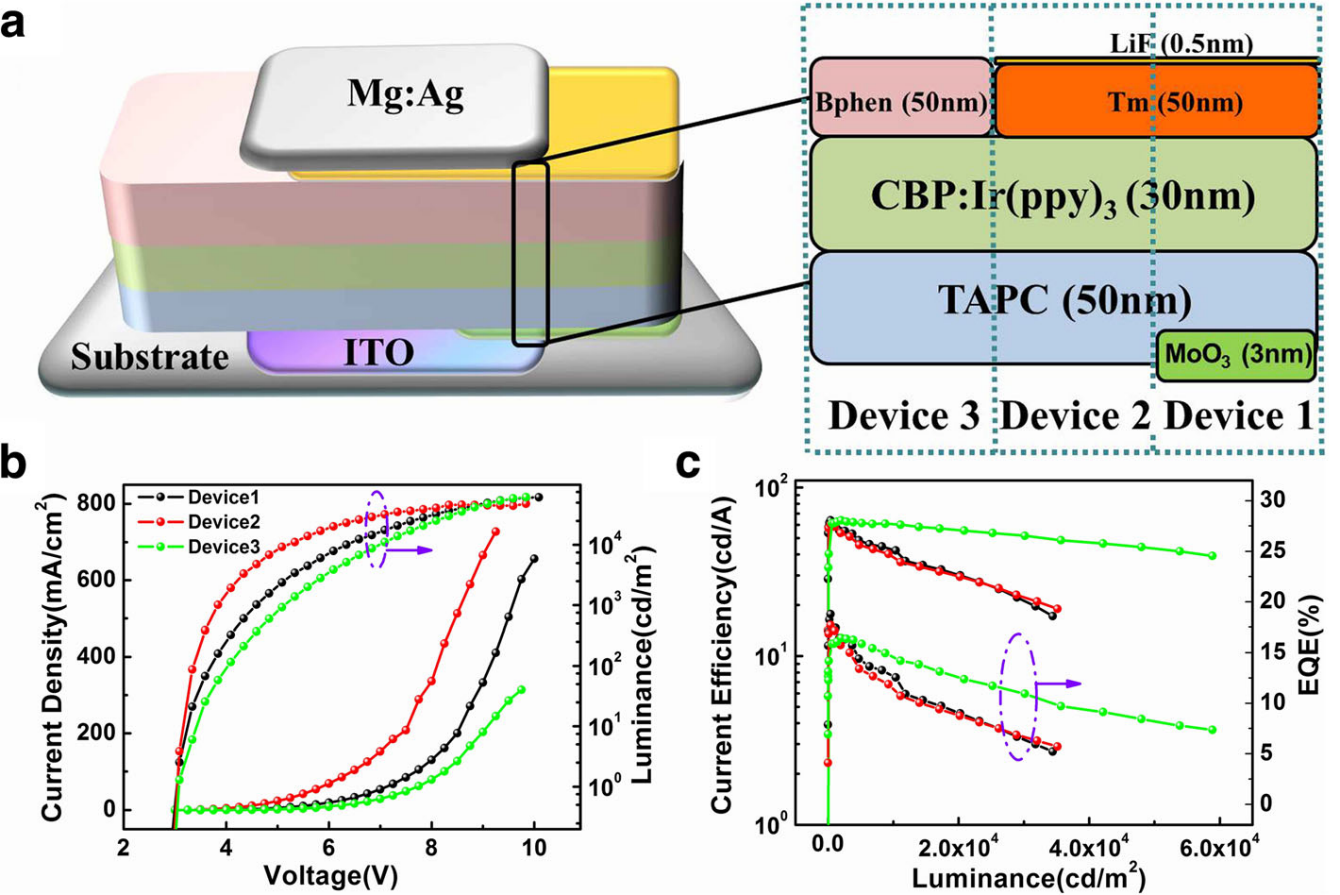
**a** Structure diagram of the three devices. **b** Current density–voltage–luminance (J-V-L) curves. **c** Current efficiency–luminance–external quantum efficiency (CE-L-EQE) curves

Finally, simple green PHOLED with a trilayer structure is obtained as shown in Fig. 3a, i.e., ITO/TAPC (50 nm)/CBP:10 wt% Ir(ppy)_3_ (30 nm)/Bphen (50 nm)/Mg:Ag (120 nm) (device 3). In addition, device 1 and device 2 have been fabricated as a contrast. The former has extra functional layers: MoO_3_ (3 nm) and LiF (0.5 nm) serving as AML and CML, respectively, while the latter only introduces a thin LiF film. Figure 3b, c shows the current density–voltage–luminance characteristics (J-V-L) and current efficiency–luminance–external quantum efficiency characteristics (CE-L-EQE) of the three devices. Although the current density and luminance of device 3 are lower than those of the other two devices as shown in Fig. 3b, the same turn-on voltage could also be observed. It indicates that the carrier injection has not been influenced by simplifying the electrode modification layers. Nevertheless, it is confused that efficiency of device 3 shows a lowest roll-off in Fig. 3c.

To explain the existence of better efficiency roll-off in device 3, we then simulate exciton-quenching mechanism via the mathematical model with the function between the EQE and J. There are two exciton-quenching mechanisms existing in PHOLEDs, i.e., triplet–triplet annihilation (TTA) and triplet–polaron annihilation (TPA). The rate equation in the model is shown as follows:

4 $$ {K}_L=\frac{q\left({\mu}_e+{\mu}_h\right)}{\varepsilon_0+{\varepsilon}_r} $$

5 $$ \frac{dn_T}{dt}={K}_L{n_P}^2-{K}_T{n}_T-\frac{1}{2}{K}_{TT}{n_T}^2-{K}_{TP}{n}_T{n}_P $$

6 $$ \frac{dn_P}{dt}=\frac{J}{qw}-{K}_L{n_P}^2 $$

7 $$ \mathrm{IQE}={K}_T{n}_T/\left(\frac{J}{qw}\right) $$

For Eq. (4), we deem that charge carriers recombine via Langevin recombination with the rate *K*_*L*_, where *q* is the elementary charge, *μ*_*e/h*_ is the mobility, *ε*_*r*_ is the relative permittivity, and *ε*_0_ is the permittivity of free space. The triplet and polaron densities, *n*_T_ and *n*_P_, were calculated by Eqs. (5) and (6), where *K*_TT_ and *K*_TP_ are the rate constants describing the kinetics of the TTA and TPA process. Actually, the internal quantum efficiency (IQE) is the ratio of radiative decaying triplets over the number of injected electrons from Eq. (7). For simplification, we do not consider light outcoupling. Moreover, the electric efficiency and the PL quantum efficiency at low current density are set to 1. Hence, the calculated IQE is used to compare with experimental EQE [33].

**Fig. 4:**
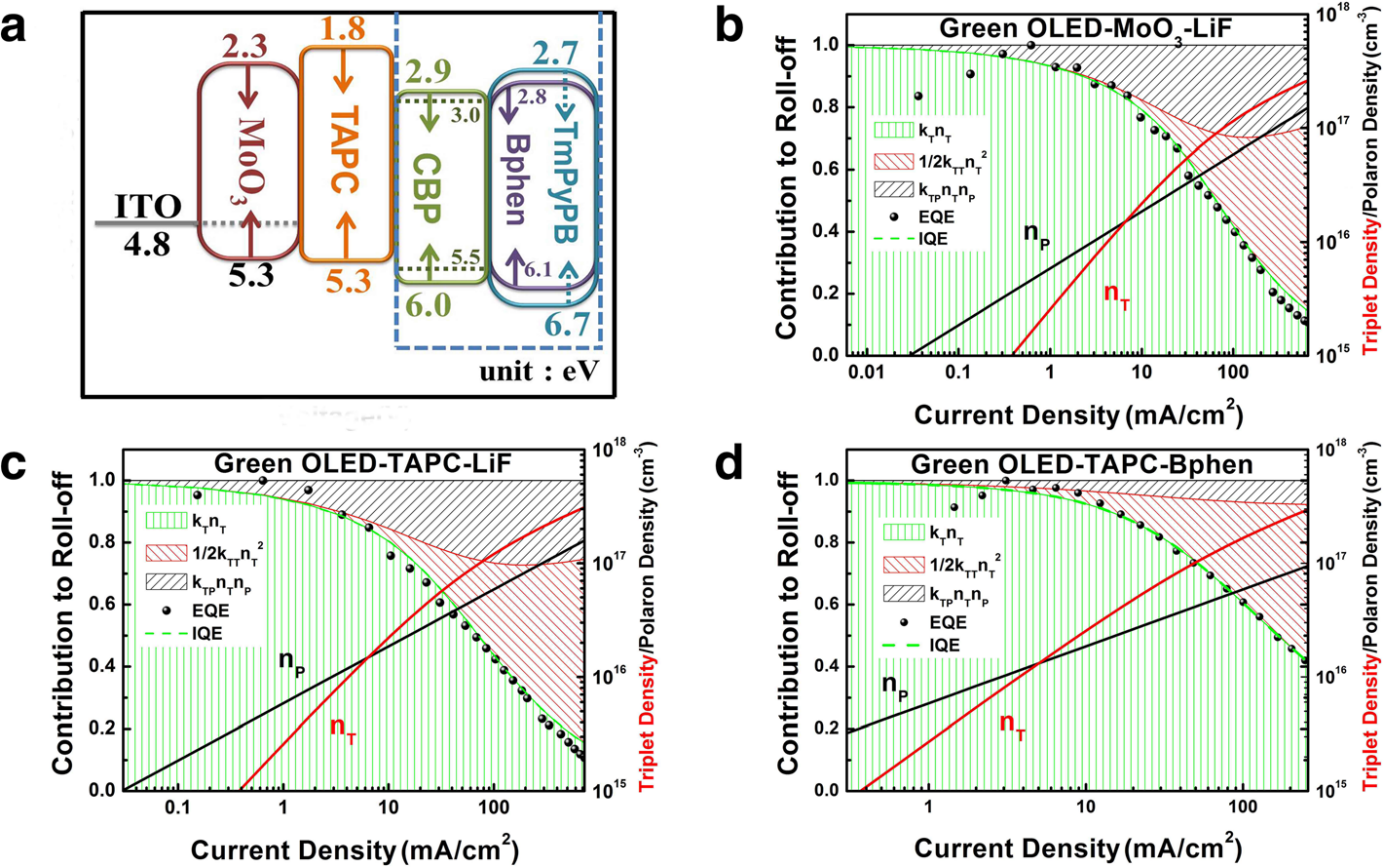
**a** Schematic energy-level diagrams of the three devices. Simulated internal quantum efficiency (IQE) (solid green or red line) and external quantum efficiency (EQE) (scattered point) act as a function of the current density. Triplet and polaron densities (red and black lines) are calculated according to Eqs. (4)–(7). Hatched areas indicate the relative contribution of TPA and TTA as well as of the emission to the overall exciton decay. **b**–**d** correspond to device 1, device 2, and device 3, respectively

As we can see from Fig. 4b–d, serious exiton-quenching effect existed in device 1 and device 2, especially TPA. CBP is bipolar transport material, but the hole mobility is an order of magnitude higher than the electron mobility. Combined with the schematic energy-level diagrams in Fig. 4a, the recombination zone should be adjacent to the interface of EML/ETL. Besides, we find that the HOMO and LUMO energy levels of Bphen are similar to those of CBP; therefore, it is easier for holes to traverse CBP layer into Bphen and few holes are accumulated at the interface between CBP and Bphen. As to device 1 and device 2, a larger energy gap between TmPyPB and CBP can also be seen in Fig. 4a, resulting in an extra hole accumulation at the interface of CBP/TmPyPB. The different hole accumulation at the interface of CBP/TmPyPB would make different influences on the excitons formed at the same interface, resulting in different TPA of devices finally.

### Analysis to the Mechanism of Exciton Recombination in Monochrome PHOLEDs

As we all know that the low concentration of phosphorescent dopant molecules leads to the long intermolecular distance, it is generally believed that phosphorescent materials act as trapping for the charge carrier. Therefore, there are two recombination mechanisms in EML of PHOLEDs, Langevin recombination I and trap-assisted recombination II. For the former, when the device is driven by applied voltage, a mass of carriers inject continuously into EML. The holes transfer through the host material, followed by an accumulation in the interface of EML/ETL. On account of a good matching to energy levels between ETL and cathode, most electrons flow through ETL up to EML and then recombine with the stored charge. In this case, excitons generated in the host material transfer to the dopant by the Förster and/or Dexter mechanisms; therefore, it belongs to the bimolecular recombination. The latter recombination zone is located in dopant due to the shallow-energy-level trapping formed by phosphorescent guest [27].

**Fig. 5:**
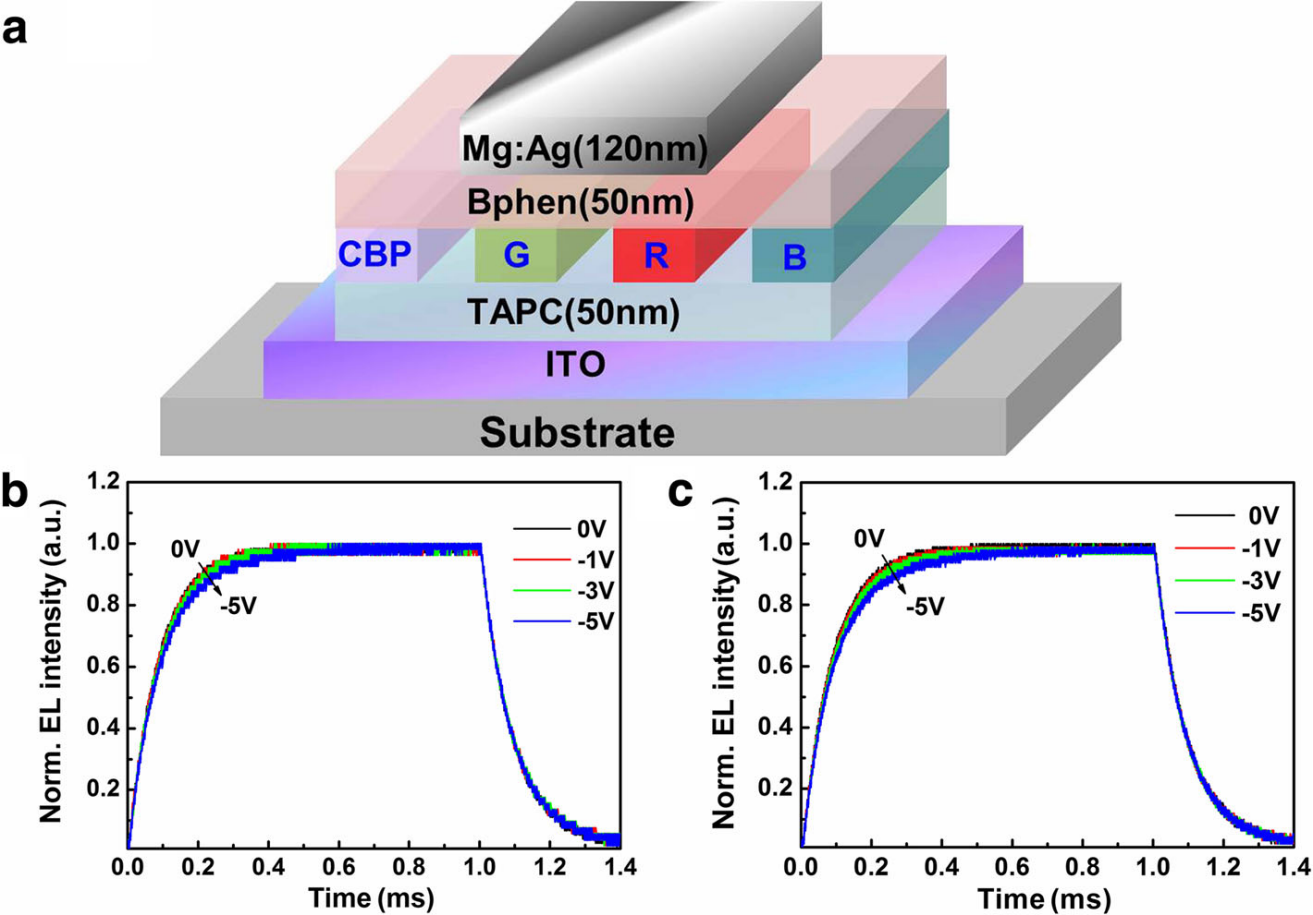
**a** Structure of devices with the different dopant in EML: pure CBP layer without dopant, CBP-doped 10 wt% Ir(ppy)_3_ (G) 5 wt% Ir(MDQ)_2_(acac) (R) and 15 wt% FIrpic (B). Normalized intensity of transient EL **b** Ir(MDQ)_2_(acac), **c** Ir(ppy)_3_ depending on the reverse bias (0 V, − 1 V, − 3 V, and − 5 V) after the applied voltage turning off .The voltage pulse width was 1 ms, and the pulse frequency was 100 Hz. A current density of 90 mA cm^−2^ was chosen to be the voltage pulse height

It is necessary to investigate the mechanisms mentioned above. As different recombination types playing a leading role in EML, it will have different impact on device performance. Structure of devices with the different dopant in EML is shown in Fig. 5a.

The recombination behaviors are investigated via the transient EL measurements. Normalized intensity of transient EL shown in Fig. 5b, c is tested by changing the reverse bias (0 V, − 1 V, − 3 V, and 5 V) after the applied voltage turning off, while the voltage pulse height corresponds to a current density of 90 mA cm^−2^. The voltage pulse width is 1 ms, and the pulse frequency is 100 Hz. As shown in Fig. 5b, c, the rise time of green and red devices slow down with the increase of reverse bias. However, this phenomenon does not occur in the other two devices. The reverse bias would take the captured carriers out of the trapping sites, and then the trapped carriers will make less contribution to EL intensity. So, we infer that trap-assisted recombination probably consists in devices fabricated by CBP-doped Ir(MDQ)_2_(acac) or Ir(ppy)_3_ due to the existence of the trapped charges [27].

**Fig. 6:**
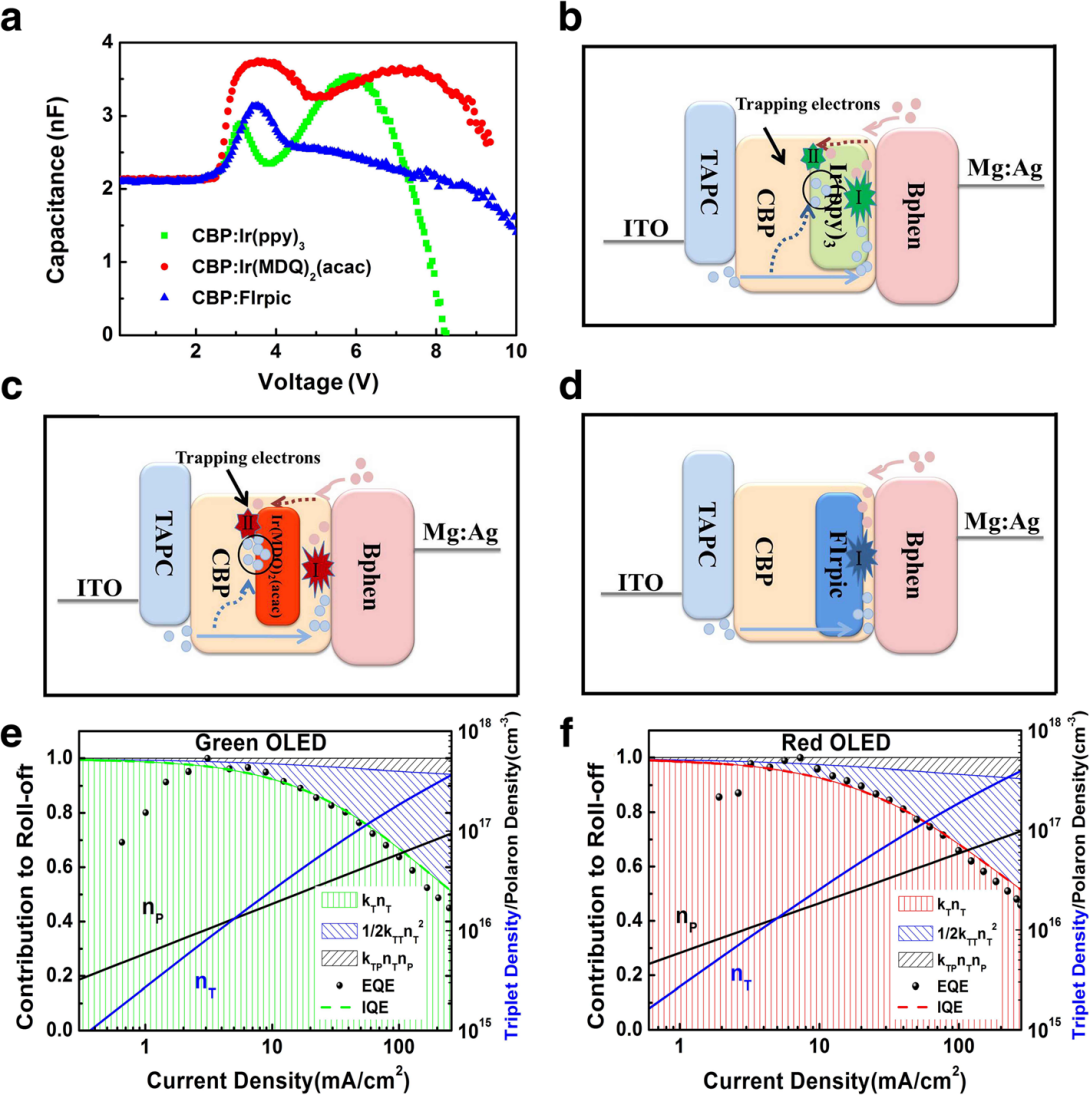
**a** C-V plots (scattered points, *f* = 1 kHz) and L-V curves (solid line) for the three different PHOLEDs-CBP-doped Ir(ppy)_3_ (green), Ir(MDQ)_2_(acac) (red), and FIrpic (blue), respectively. The purple dashed lines marked in the graph representing the *V*_on_. Where the arrows line point are the inflection points in the curves. Schematic energy-level diagrams on an ITO/TAPC (50 nm)/CBP:10 wt% × (30 nm)/Bphen (50 nm)/Mg:Ag (120 nm) OLED. The X is **b** Ir(ppy)_3_ (green), **c** Ir(MDQ)_2_(acac) (red), and **d** FIrpic (blue). Two recombination mechanisms occur probably in EML, marked as I (Langevin recombination) and II (trap-assisted recombination). Additionally, the blue point represents hole, while the pink point is electron. **e** Green phosphorescent OLED based on CBP:Ir(ppy)_3_. **f** Red phosphorescent OLED based on CBP:Ir(MDQ)_2_(acac). Triplet and polaron densities (blue and black lines) are calculated according to Eqs. (4)–(7)

Further study of the existence of trapped charges is developed by impedance spectroscopy measurement with the result of capacitance–voltage curves shown in Fig. 6a. Two strong peaks could be observed in the C-V characteristics of green and red devices. Moreover, there is only one apparent peak in the blue device. The bias voltage corresponding to the first peak of the three devices is almost identical to the turn-on voltage. It can be interpreted that charge carriers inject constantly into devices when devices start to be driven by the applied voltage, resulting in the increase of capacitance at low voltage. And then for the green device, we deem that a small amount of the injected holes are captured by trapping via phosphorescent dye. Subsequently, they are recombined with electrons from cathode causing the trap-assisted recombination. Hence, parts of these accumulated charges begin to reduce at approximately 3 V. Similar phenomenon can be seen in the C-V curve of the red device, the falling of the first peak at 3.5 V is caused by trap-assisted recombination. In addition, the higher peak of C-V curve from 2.5 to 5 V can attribute to the stronger trapping effect in the red device.

More holes inject with the augment of applied voltage; besides the trapped ones, most of them get to store at the interface of EML/Bphen. Therefore, both of the C-V curves of green and red devices rise again. At this point, the Langevin recombination has happened in the EML causing the reduction of internal stored carriers. When the dissipative rate of charges exceeds their injection rates, the accumulated charges reduce rapidly and the C-V curve exhibits a sharp drop. The recombination process is shown in Fig. 6b, c. For comparison, only one strong peak appears in the capacitance characterize of the blue device, indicating that only the Langevin recombination occurs in the EML. Schematic energy-level diagrams with the recombination mechanism are shown in Fig. 6d.

We can also verify our results via the mathematical model mentioned above. It is well known that TTA is caused by high triplet density, while the high Langevin recombination rate would reduce the triplet density. So, the TTA can be associated with the Langevin recombination. TPA depends on the charge trapping characteristics of the host–guest system: when the emitter molecules constitute a trapping site for polarons within the host, accelerated TPA can be expected [33].

The corresponding contribution of TTA and TPA to the overall annihilation for the two devices with the EML of CBP:Ir(ppy)_3_ and CBP:Ir(MDQ)_2_(acac) is shown in Fig. 6e, f. The calculated IQE is coincident to the measured EQE; moreover, the distinction between IQE and EQE curves at low bias voltage is caused by leak current. For the two devices, the polaron density is larger than the triplet density when the current density is below 5 mA cm^−2^. Therefore, we believe that there are two quenching processes on operation condition, meaning that two recombination types occur in the EML. A higher percentage of TPA occurs in the red device, reflecting the stronger trap-assisted recombination [33, 34].

In terms of the quenching process discussed above, it is obvious that TTA and TPA may dramatically decrease the efficiency of phosphorescent OLEDs. Therefore, in order to research the effect on device performance by changing host material, we prepare red devices with different hosts, i.e., CBP, TCTA, 2,6-bis(3-(carbazol 9,9′-[4′-(2-ethyl-1*H*-benzimidazol-1-yl)-9-yl) phenyl)pyridine [26DCzPPy] and 2,2′[2″-1,3,5-benzinetriyl)-tris(1-phenyl-1-H-benzimidazole) [TPBi]. When CBP is used as the host, the TTA and TPA are efficiently limited. Therefore, the CBP is chose to act as the host in this work.

### Single-Layer White OLEDs

**Fig. 7:**
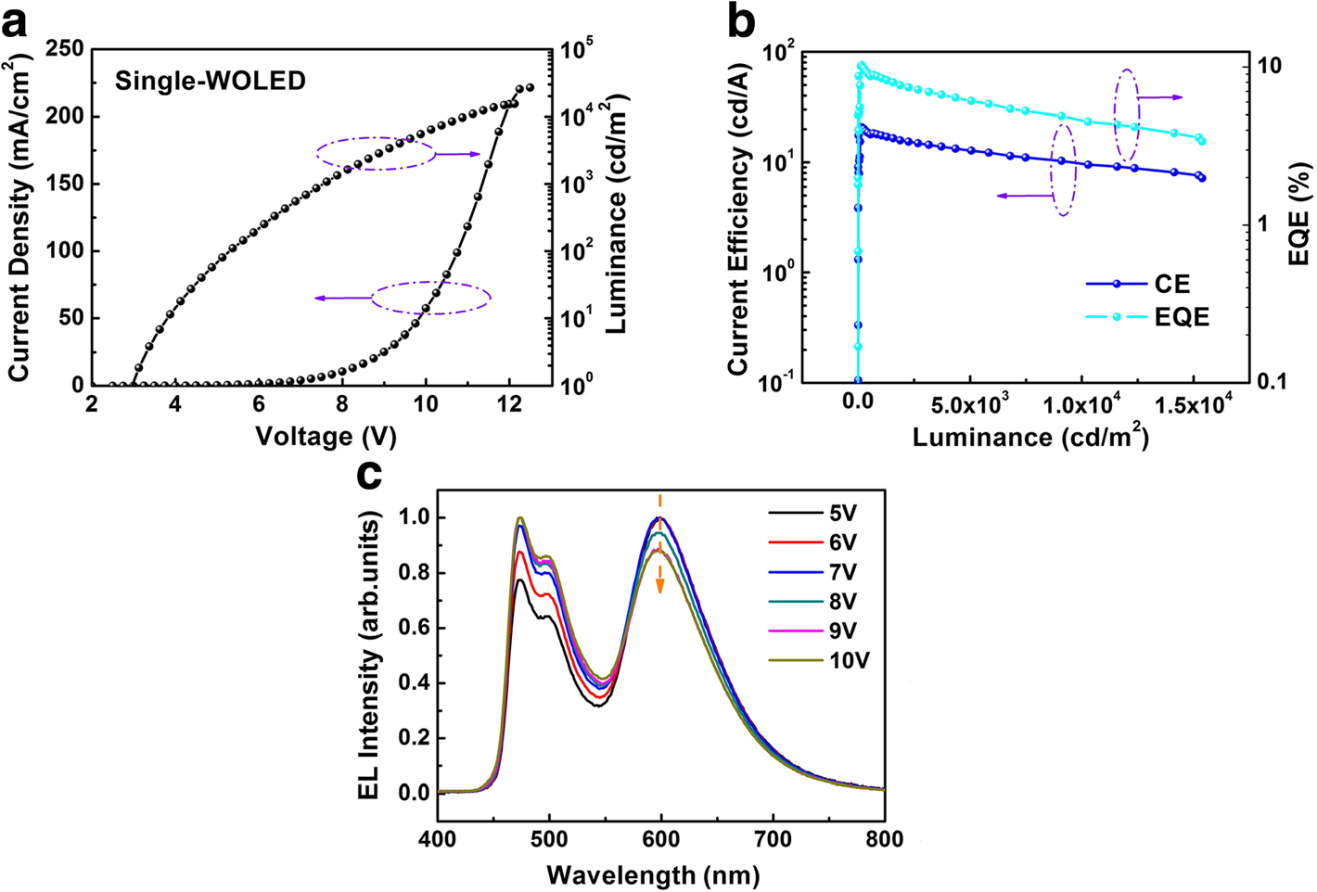
**a** Current density–voltage–luminance (J-V-L) curves of the WOLEDs. **b** Current efficiency–luminance–external quantum efficiency (CE-L-EQE) curves. **c** Normalized EL spectra of the white OLEDs. The orange arrow shows the weakened spectra versus applied voltage

Finally, we also fabricate trilayer WOLEDs with the structures of ITO/TAPC (50 nm)/CBP:FIrpic:Ir(MDQ)_2_(acac) (3:1:0.01) (30 nm)/Bphen(50 nm)/Mg:Ag (120 nm). Figure 7a shows the current density–voltage–luminance (J-V-L) characteristic of the device. It indicates that our single-EML WOLEDs possess a low turn-on voltage below 3 V. Moreover, we achieve a high current efficiency of 21 cd A^−1^. Normalized EL spectra of the device in Fig. 7c show that the red intensity tends to be weakened when the bias voltage increases from 5 to 9 V. It should be attributed to that the trapping effect of the red dye molecule merely plays a major role under low bias voltage. At a practical luminance of 5840 cd m^−2^, the CIE coordinates of devices are (0.39, 0.39), corresponding to warmish-white emission.

## Conclusions

In summary, efficient phosphorescent OLEDs have been prepared based on a simple trilayer structure (TAPC/EML/Bphen). We simplify the devices gradually via impedance spectroscopy and transient measurement. The EL performances of trilayer devices could be still comparable to the conventional devices with modification layers. Langevin recombination and trap-assisted recombination are certified to be existed in red and green phosphorescent devices by capacitance–voltage measurement. In addition, mathematical model is used to describe the TTA and TPA quenching processes, which are relevant to the two recombination types mentioned above. Based on the above analysis, we obtain the efficient WOLEDs with low roll-off. These results demonstrate an effective approach towards simplified OLED with high efficient and low cost.

## Abbreviations

26DCzPPy: 2,6-Bis(3-(carbazol 9,9′-[4′-(2-ethyl-1H-benzimidazol-1-yl)-9-yl) phenyl) pyridine
AML: Anode modification layer
Bphen: 4,7-Diphenyl-1,10-phenanthroline
C: Capacitance
CBP: 4,4′-*N*,*N*′-Dicarbazole-biphenyl
CE-L-EQE: Current efficiency-luminance-external quantum efficiency
CML: Cathode modification layer
C-V: Capacitance–voltage
C-V-L: Capacitance–voltage–luminance
EBL: Electron-blocking layer
EL: Electroluminescence
EML: Emitting layer
EQE: External quantum efficiency
ETL: Electron-transporting layers
FIrpic: Bis [(4,6-difluorophenyl)-pyridinato-N,C^2^′] (picolinato) Ir(III)
HBL: Hole-blocking layer
HOMO: Highest occupied molecular orbital
HTL: Hole-transporting layers
IQE: Internal quantum efficiency
Ir(MDQ)_2_(acac): Iridium (III) bis-(2-methyldibenzo-[f, h] quinoxaline) (acetylacetonate)
Ir(ppy)_3_: Tris(2-phenylpyridine) iridium;
IS: Impedance spectroscopy
ITO: Indium tin oxide
J-V: Current density–voltage
J-V-L: Current density–voltage–luminance
LUMO: Lowest unoccupied molecular orbital
OLEDs: Organic light-emitting devices
PEDOT:PSS: Poly(3,4-ethylenedioxythiophene)-poly(styrene sulfonate)
PHWOLEDs: Phosphorescent white OLEDs
TAPC: Di-[4-(*N*,*N*-ditolyl-amino)-phenyl] cyclohexane
TCTA: 4,4′,4″-Tris (carbazol-9-yl)-triphenylamine
TmPyPB: 1,3,5-Tri(m-pyrid-3-yl-phenyl) benzene
TPA: Triplet-polaron annihilation
TPBi: 2,2′[2″-1,3,5-Benzinetriyl)-tris(1-phenyl-1-H-benzimidazole)
TTA: Triplet-triplet annihilation
